# Cost effectiveness of different treatment strategies with natalizumab for pregnant women with multiple sclerosis

**DOI:** 10.1007/s00415-024-12736-z

**Published:** 2025-01-07

**Authors:** Magdalena Walbaum, Anushka Madhukar, Ruth Dobson, Eva Cyhlarova, Laura Castro-Aldrete, Antonella Santuccione Chadha, Martin Knapp

**Affiliations:** 1 Care Policy and Evaluation Centre, London School of Economics and Political Science, London, WC2A 2AE UK; 2 Centre for Preventive Neurology, Wolfson Institute of Population Health, Queen Mary University London, London, UK; 3 Department of Neurology, Royal London Hospital, Barts Health NHS Trust, London, UK; 4 Women’s Brain Foundation, Zurich, Switzerland

**Keywords:** Multiple sclerosis, Cost-effectiveness, Treatment strategies, Natalizumab, Pregnancy

## Abstract

**Background:**

The management of multiple sclerosis (MS) during pregnancy poses significant challenges. This study aimed to evaluate the cost-effectiveness of three natalizumab treatment strategies during pregnancy from the UK healthcare system’s perspective.

**Methods:**

A Markov model was developed to assess the health outcomes and costs associated with three treatment strategies: continuous natalizumab treatment throughout pregnancy, treatment until the first trimester followed by discontinuation, and discontinuation at conception with resumption post-pregnancy. The model incorporated data on relapse rates, disability progression, costs and quality-adjusted life years (QALYs). Sensitivity analyses were conducted.

**Results:**

Continuing natalizumab throughout pregnancy was the most cost-effective strategy, yielding the highest incremental QALY gains and the lowest incremental cost per QALY (£1713 per QALY), with a net monetary benefit of £743. The sensitivity analyses confirmed the robustness of these findings and the use of generic or biosimilar forms of natalizumab further reinforced the cost-effectiveness of continuous treatment, with the biosimilar option proving cost-saving.

**Conclusion:**

Continuing natalizumab treatment throughout pregnancy is the most cost-effective approach for managing MS in pregnant women. These findings should inform clinical guidelines and support healthcare providers and women with MS planning their family in making evidence-based decisions to improve the management of MS during pregnancy.

**Supplementary Information:**

The online version contains supplementary material available at 10.1007/s00415-024-12736-z.

## Background

Multiple sclerosis (MS) is a chronic inflammatory disease characterised by demyelination affecting the central nervous system [1, 2]. The age at MS onset is variable, with peak incidence between the ages of 20 and 40 [3, 4]. There is a significant gender disparity, with MS being up to three times more common in females [5, 6]. The majority of people with the disease are initially diagnosed with relapsing remitting MS (RRMS), with episodes of relapse, followed by periods of stability [7]. These relapses can cause irreversible damage resulting in long-term disability [8]. Although incurable, several disease-modifying treatments (DMTs) are available for the treatment of MS [9]. DMTs aim to reduce the frequency and severity of relapses, slow down disability progression, and improve overall quality of life. The selection and initiation of DMTs for individual patients depend on multiple factors such as disease activity, individual patient characteristics, and potential side-effects.

Women with MS may face difficult decisions regarding balancing DMT use and family planning [10]. Approximately, one in three women with MS becomes pregnant after diagnosis [11]. Pregnancy affects the course of MS in women, with significant changes in relapse rates during the pregnancy and post-partum periods, with DMT strategy additionally impacting on pre-conception relapse risk. The historical studies have shown that annualised relapse rates (ARR) decrease after the first trimester compared to pre-conception levels, with a significant increase in the 3 months after delivery (post-partum rebound) and return to pre-pregnancy rates within 4–6 months postpartum [12]. More recent work has shown that the post-partum rebound appears to be less marked, possibly reflecting either improved DMT algorithms around pregnancy, or extension of diagnostic criteria resulting in milder disease at pregnancy [13]. In the post-partum period, breastfeeding is safe and recommended for women with MS [14–16]. The risk of post-partum relapses appears to reduce significantly with breastfeeding [17, 18], which is associated with 37% lower risk of postpartum relapse on average compared to not breastfeeding [19], although selection bias and reverse causation may impact on these observational study findings.

Women of childbearing age require consideration when selecting treatment regimes, as some DMTs may affect fertility (haemopoietic stem cell transplantation, HSCT), be associated with congenital malformations (fingolimod) [20], or pose risks associated with withdrawal rebound [14], with potential long-term consequences [21]. Consequently, women with MS naturally have concerns about family planning including hesitation regarding use of DMTs during pregnancy or breastfeeding, as well as about managing childcare while experiencing relapses [22]. Historically, women have often faced a choice between treatment plans and family planning and some were advised to begin treatment after pregnancy [17].

Whilst evidence regarding the safety profiles of DMTs when used in and around pregnancy has increased substantially in the last decade, research regarding the impact of DMT withdrawal has lagged behind. Recent studies have used drug trials to compare the effectiveness and risks of various DMTs, but there is little evidence regarding the advantages and disadvantages of stopping versus continuing treatment. The relapses resulting from withdrawal of treatment can cause substantial long-term morbidity [14], yet the impact of this beyond individual level has not been assessed. For example, UK consensus on pregnancy in multiple sclerosis strongly suggests continuing treatment with natalizumab during pregnancy given the significant risk of disease reactivation and/or rebound on stopping treatment [17]. The analysis described here takes the case of the use of natalizumab to investigate the cost-effectiveness of three treatment strategies for women during pregnancy from the perspective of the UK healthcare system. We analyse the potential risks and challenges associated with treatment discontinuation at, during and after pregnancy.

## Methods

### Study design

We conducted cost-effectiveness analysis to estimate the health and economic impacts of continuing or discontinuing natalizumab treatment for women during pregnancy in the UK from the healthcare system perspective. The model was constructed using Microsoft Excel and the analysis was reported according to the Consolidated Health Economic Evaluation Reporting Standards (CHEERS) statement [23].

### Model structure

**Fig. 1:**
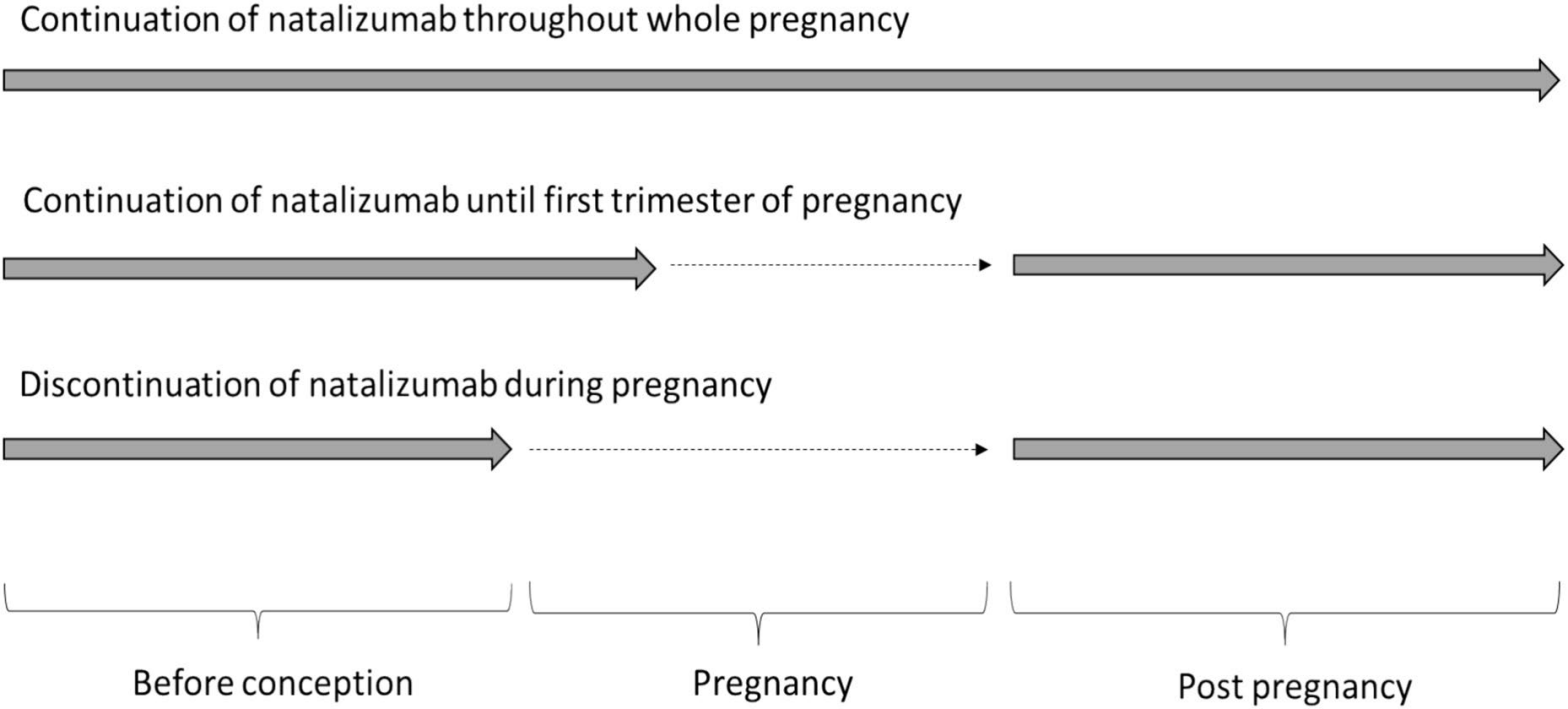
Treatment strategies of natalizumab

We developed a Markov cohort simulation model to assess health outcomes and costs associated with different patterns of natalizumab use for MS at conception and during and after pregnancy (Fig. 1). We compared three treatment strategies: (i) continuous drug treatment throughout pregnancy; (ii) continuous treatment until the first trimester then discontinuation; and (iii) discontinuation at conception and resuming treatment post-pregnancy; reflecting the practice of giving no further infusions following the first positive pregnancy test. For simplicity, this last strategy has been referred as stopping at conception throughout the rest of the text. This analytical approach was discussed and iteratively revised with an advisory group of experts in the MS field.

We structured our model into monthly cycles, beginning 12 months prior to conception, including 9 months of pregnancy and 12 months post-pregnancy, resulting in a total time horizon of 33 months. The starting point for the model was 12 months prior to conception. The probability to transition between cycles in the model was set to 1, indicating that every woman progresses from one monthly cycle to the next. This setup reflects the natural monthly progression throughout pregnancy. We extracted data from published studies to populate the model (Table 1). The costs and quality-adjusted life years (QALYs) were calculated independently for each cycle, and total QALYs and costs were calculated by summing all cycle totals. The model included costs such as acquisition and monthly administration of the drug, costs related to relapse (cost per event), and direct medical costs related to the different levels of the Expanded Disability Status Scale (EDSS, Table 2) [24]. No data on neonatal outcomes were included, as there is no evidence indicating an increased risk of adverse outcomes with the use of natalizumab during pregnancy [17, 21].

**Table 1:** Inputs to the model

	Discontinuation at conception	Discontinuation after first trimester	Continuation through pregnancy	Source
ARR pre-conception	0.29	0.29	0.21	Yeh 2021
ARR pregnancy	0.5–0.6	0.2–0.5	0.2	Yeh 2021
ARR post-pregnancy	0.4–0.9	0.3–1	0.2–0.6	Yeh 2021
EDSS progression	21%	16%	12%	Hellwig 2022^a^
Drug cycles	6	6.75	8.25	Calculation^b^
EDSS pre-pregnancy				
0–1	53.9%			Yeh 2021
2–3	35.6%			
4 +	5.9%			
Disutility relapse^c^	– 0.07			Orme 2007
Utility by EDSS				
0–1	0.88			Van Eijndhoven 2020
2–3	0.75			
4 +	0.43			

Hellwig et al. 2022 only reported the proportion of EDSS progression for discontinuation or discontinuation after the first trimester

*ARR* annualised relapse rate, *EDSS* Expanded Disability Status Scale

^a^We calculated the proportion of progression assuming a linear relationship, using the equation *y* = – 4.58 *x* + 25.63

^b^We calculated the total cycles of drug use of the 33-months period assuming a frequency of a cycle every 4 weeks

^c^Disutility of relapse was derived from Orme et al. 2007, estimated using Quality adjusted life years (QALYs)

**Table 2:** Costs included in the model

	Costs (£)	Source
Drug acquisition^a^		
Tysabri^®^	£1130	Spelman 2022
Generic form	£870	Expert opinion
Biosimilar	£666	Expert opinion
Administration costs^b^	£223	Spelman 2022
Cost per relapse	£1623	Tyas 2007
Cost per EDSS		
0	£488	Spelman 2022
1	£887	
2	£4611	
3	£3656	
4	£3474	
5	£4850	
6	£9602	
7	£15,412	
8	£27,786	
9	£35,545	

^a^Costs of acquisition of 300 mg vial. We assumed one vial every 4 weeks

^b^Unit cost of administration. We assumed IV infusion every 4 weeks to estimate the total costs of administration

The analysis followed National Institute for Health and Care Excellence (NICE) recommendations, taking a health sector perspective, with effectiveness assessed in terms of QALYs. Incremental cost-effectiveness ratios (ICER) were calculated, defined as difference in mean costs between treatment strategies (∆C), divided by difference in mean outcomes (∆E). An intervention can be interpreted as representing value for money if the ICER is below a threshold of willingness to pay (WTP) for a unit of additional effectiveness $\lambda$ [25]. This decision rule that can be expressed as:

1 $$\Delta C/\Delta E \, < \lambda$$

We also examined the incremental net monetary benefit (NMB) [25]. NMB can be expressed as a rearrangement of the decision rule in (1):

2 $$\lambda *\Delta E - \Delta C > 0$$

This represents the monetary value of gains in outcomes attributed to the treatment at a particular WTP, minus the additional treatment costs [26]. In England, NICE uses a WTP threshold of £20,000 per QALY as the lower bound for health sector cost-effectiveness. The costs and QALYs were discounted at an annual rate of 3.5% following NICE guidelines. The costs were adjusted to 2024 prices (GBP) using the Consumer Price Inflation (CPI) index for health [27].

### Model inputs

The model inputs and assumptions used are drawn from published literature on the use of natalizumab in pregnancy (Table 1). Additional unpublished data on costs of drug acquisition in generic form and for biosimilars of natalizumab were obtained from expert opinion and own calculations when needed (Table 1). We calculated the total drug cycles that would need to be administered over the 33-month period, assuming a frequency of one cycle every 4 weeks. Annualised relapse rates (ARR) before conception, during pregnancy and post-pregnancy were extracted from Yeh et al. [28]. These rates are visually represented in the supplementary material of that study; we extracted the estimates from the figure using PlotDigitizer [29].

The disability progression rates, based on EDSS categories, were extracted from Hellwig et al. [30]. This study examined disability progression in women who discontinued treatment at conception and those who discontinued after the first trimester. To calculate the disability progression rate of continuing treatment throughout pregnancy, we interpolated linearly between these two estimates. Proportions of women with MS with different levels of disability, based on EDSS categorisation, were extracted from Yeh et al. [28]. QALY loss per relapse was sourced from Orme et al. [31]. QALYs were categorised by EDSS levels and derived from Van Eijndhoven et al. [32].

Data on the costs of relapse were extracted from Tyas et al. [33] and consider direct medical costs related to the event. The costs of disability per EDSS level were sourced from Spelman et al. [24] and Thompson et al. [34]. These costs consider health care costs (inpatient and outpatient costs) and community care services related to each EDSS level. The costs of drug acquisition were extracted from Spelman et al. [24] and Tyas et al. [33] and expert opinion (Table 2). Total costs of drug consider costs of acquisition of 300 mg vial, assuming one vial every 4 weeks, and costs of administration, assuming intravenous (IV) infusion every 4 weeks.

### Sensitivity analysis

Probabilistic sensitivity analysis was performed with 1000 simulations to account for variations in base case model inputs: (i) different ARR during and after pregnancy across the three treatment patterns; (ii) varying costs related to relapse and disability; and (iii) assuming a WTP threshold of £30,000. For points (i) and (ii), adjustments were made by – 20% to + 20% for ARR estimates and costs associated with each treatment pattern, assuming beta distribution for proportions and gamma distribution for cost variations [35, 36]. The results of the PSA were expressed in a cost-effectiveness plane and cost-effectiveness acceptability curve. In addition, we conducted scenario analyses assuming different EDSS progression rates, using a logarithmic function rather than the linear function used in the base case, and different drug prices (including generic and biosimilar prices).

## Results

We present total costs and QALYs associated with the three different treatment patterns of natalizumab in Table 3. In the scenario where natalizumab was discontinued at conception, the total costs were £19,727 per person. The total QALYs for discontinuation of natalizumab at conception was 1.9 per person over the 33-month period. In the case of discontinuation of natalizumab after the first trimester, per-person costs were £22,607. The total QALYs for discontinuation of natalizumab after the first trimester was 1.92 per person. For individuals who continued natalizumab throughout pregnancy, the total costs were £19,796 per person. The total QALYs of continuation of natalizumab throughout pregnancy was 1.94 per person.

**Table 3:** Costs and QALYs associated with different treatment patterns of high-efficacy DMT during pregnancy (per person)—Base case

Treatment strategy	Total costs (£)	Total QALY
Discontinuation at conception	19,727	1.90
Discontinuation after first trimester	22,607	1.92
Continuation through pregnancy	19,796	1.94

^a^Base case considered costs related to Tysabri^®^ use. Costs and QALY loss estimated over the 33-month period

**Fig. 2:**
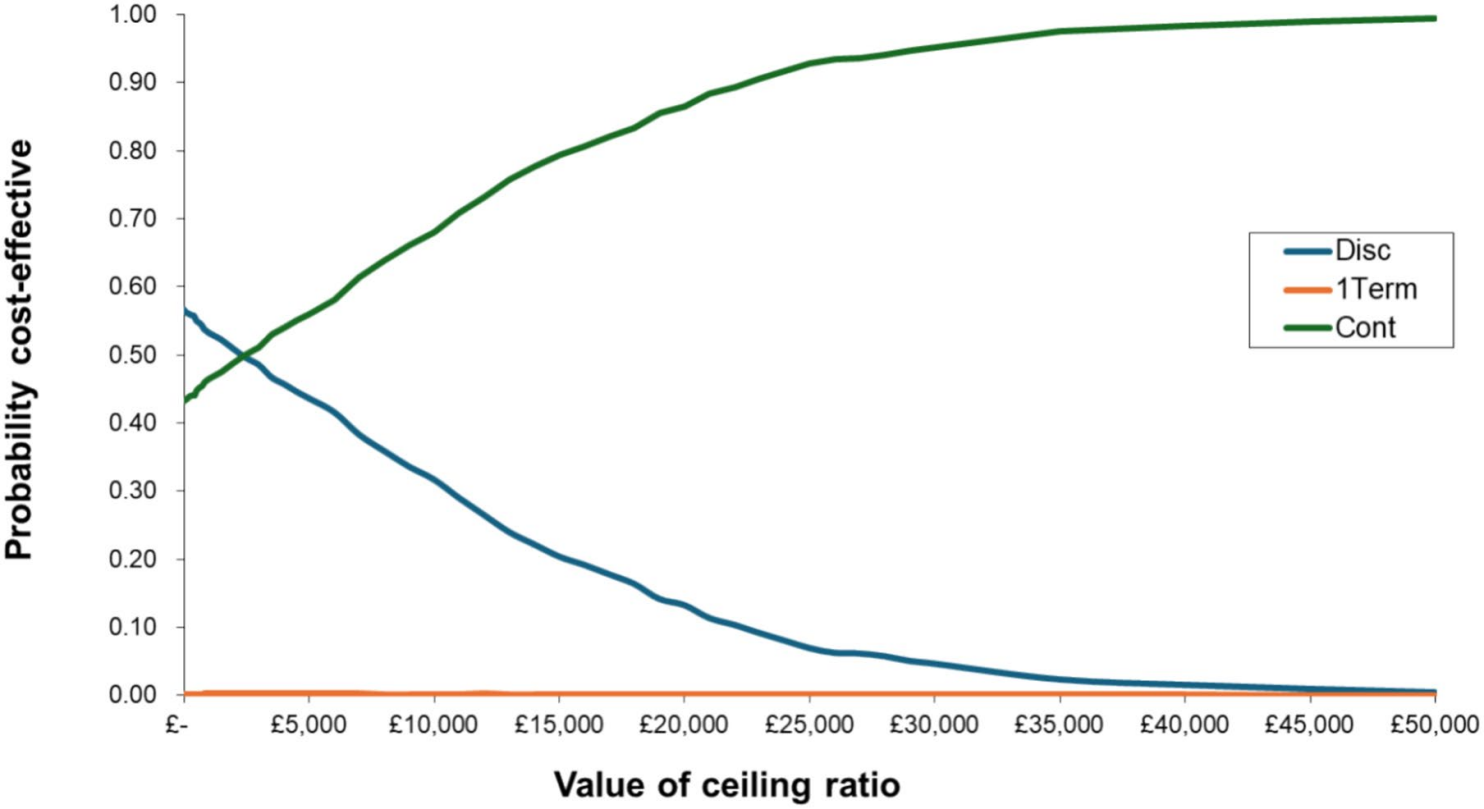
Cost effectiveness acceptability curves

Continuing treatment with natalizumab through pregnancy is more effective but more costly compared to discontinuing treatment at conception; however, it is cost-effective by reference to NICE. It yields the highest incremental QALY gains (0.04) at a lower incremental cost (£70), resulting in the most favourable ICER (£1713 per additional QALY) and the highest net monetary benefit (£743). Discontinuing natalizumab after the first trimester is not cost-effective compared to discontinuing treatment at conception, with an ICER of £159,067 per QALY and a NMB of – £2518 (Table 4). Uncertainty around these estimates is illustrated in the cost-effectiveness acceptability curve by plotting the probability that the intervention is cost-effective as the WTP threshold increases (Fig. 2). Simulating cost-effectiveness with different WTP thresholds indicated that continuing treatment during pregnancy has 0.865 probability of being cost-effective at the NICE threshold WTP of £20,000 per QALY, and probability of 0.952 of being cost-effective at WTP of £30,000 per QALY, reflecting greater confidence that the benefits of continuing treatment during pregnancy outweigh the costs associated with ongoing treatment.

**Table 4:** Incremental costs and QALYs—Base case

Treatment strategy	Incremental cost (£)^a^	Incremental QALY^a^	ICER	NMB (£)
Discontinuation at conception	–	–	–	–
Discontinuation after first trimester	2880	0.02	159,067	– 2518
Continuation through pregnancy	70	0.04	1713	743

^a^Incremental costs and QALYs shown as the difference between the treatment strategy and discontinuation of natalizumab

The results of the incremental costs and QALYs, considering costs of generic form of natalizumab and biosimilar, are presented in Tables 5 and 6. These results are consistent with the base case analysis, where discontinuation after the first trimester and continuation through pregnancy were cost-effective compared to discontinuation at conception. In both scenarios, using biosimilar or generic forms of natalizumab, the continuation of treatment through pregnancy was cost-saving and more effective, hence considered dominant over discontinuation at conception.

**Table 5:** Incremental costs and QALYs—generic form of natalizumab

Treatment strategy	Incremental cost (£)^a^	Incremental QALY^a^	NMB (£)
Discontinuation at conception	–	–	–
Discontinuation after first trimester	2547	0.02	– 2183
Continuation through pregnancy	– 905	0.04	1724

^a^Incremental costs and QALYs shown as the difference between the treatment strategy and discontinuation of natalizumab

**Table 6:** Incremental costs and QALYs—biosimilar of natalizumab

Treatment strategy	Incremental cost (£)^a^	Incremental QALY^a^	NMB (£)
Discontinuation at conception	–	–	–
Discontinuation after first trimester	2475	0.02	– 2120
Continuation through pregnancy	– 1356	0.04	2162

^a^Incremental costs and QALYs shown as the difference between the treatment strategy and discontinuation of natalizumab

## Discussion

This study investigates the cost-effectiveness of three natalizumab treatment strategies for women with MS during pregnancy from the perspective of the UK healthcare system, based on available data and current UK recommendations [17]. The strategies examined include: (i) continuous natalizumab treatment throughout pregnancy, (ii) continuous treatment until the first trimester and then discontinuation, and (iii) discontinuation at conception and resuming post-pregnancy. The cost effectiveness associated with both originator product and biosimilars were considered, making these results relevant and robust to changes related to the use of natalizumab for the treatment of pregnant women with multiple sclerosis in the UK. The analysis employed a Markov cohort simulation model to assess health outcomes and costs associated with these treatment patterns.

The results demonstrate that continuous natalizumab treatment throughout pregnancy is the most cost-effective strategy. This approach yields the highest incremental QALY gains and the lowest incremental cost per QALY, resulting in the most favourable ICER and highest NMB. Specifically, continuing treatment throughout pregnancy resulted in an ICER of £1713 per QALY and an NMB of £743 compared to discontinuing treatment at conception. Discontinuing natalizumab at conception or after the first trimester increases the costs per person due to a higher risk of relapses, disability progression, and loss of QALYs, compared to continuing treatment through pregnancy, even when factoring in the costs of continuing to take the drug. These findings can provide valuable insight into treatment patterns and help women with MS make informed decisions during pregnancy, as well as informing healthcare policy within a nationally funded system.

Several studies indicate that women with RRMS who continue natalizumab treatment through pregnancy experienced fewer relapses during the first trimester compared to those who discontinue treatment [30], thus those discontinuing treatment incur higher healthcare costs. The evidence shows that natalizumab exposure during pregnancy does not significantly increase the risk of adverse pregnancy outcomes or foetal malformations [21]. The meta-analysis and population-based studies indicate that breastfeeding can lower relapse rates among new mothers. The bioavailability of natalizumab in breastmilk is negligible, which should alleviate concerns regarding the risk of transfer to infants [16]. This evidence supports the safe continuation of natalizumab throughout pregnancy and into the post-partum period, mitigating the risk of rebound [21].

In our analysis, we assumed no washout period when considering the continuation or discontinuation of natalizumab during pregnancy. This decision was based on two key factors. First, we recognise the variability in the time it takes for women to conceive and in clinical practice regarding treatment cessation prior to pregnancy. However, the evidence indicates that the median washout period has significantly shortened over time, from 12 months before 2005 to 0 months after 2011 [28], reflecting changes in treatment practices. Second, we adopted a conservative approach by focusing only on differences in treatment schemes during pregnancy, rather than accounting for discontinuation of natalizumab on the preconception period, to avoid introducing variability that could skew the data. Moreover, we acknowledge that natalizumab dosing regimens vary in clinical practice; however, we could not fully capture the individualised nature of dosing schemes within the scope of our model. To partially address this variability, we included probabilistic sensitivity analysis to account for variations in dosing regimens and their potential impact on costs and outcomes. Nonetheless, we recognise that further individual simulation models could be developed to fully capture these differences in clinical practice.

The sensitivity analyses confirmed the robustness of the findings, indicating that varying key parameters such as ARR, disability progression rates, and drug costs did not significantly alter the results. The probabilistic sensitivity analysis, which accounted for parameter uncertainty through 1000 simulations, consistently showed that continuing natalizumab treatment during pregnancy remained the most cost-effective strategy. Additionally, scenario analyses considering the costs of generic and biosimilar forms of natalizumab yielded results consistent with the base case. Specifically, both the generic and biosimilar forms of natalizumab showed that continuing treatment throughout pregnancy remained the most cost-effective strategy compared to discontinuation at conception or after the first trimester. In particular, continuing treatment throughout pregnancy with the biosimilar form of natalizumab not only proved to be more cost-effective but also cost saving, underscoring the significant implications for healthcare policy and the management of multiple sclerosis in pregnant women.

These findings have significant implications for clinical practice and healthcare policy. The evidence supports the continuation of natalizumab treatment during pregnancy for women with MS, potentially informing guidelines and recommendations. The healthcare providers should consider these results when advising women with MS on treatment options during pregnancy, balancing the benefits of reduced relapse rates and disability progression against the costs of continuous natalizumab treatment. Furthermore, the study highlights the need for personalised treatment plans and close monitoring of women with MS during pregnancy to optimise health outcomes for both the mother and child, emphasising the importance of individualised treatment approaches for pregnant women with MS to minimise relapse risks and adverse pregnancy outcomes.

## Conclusions

There is strong evidence that continuing natalizumab treatment throughout pregnancy is the most cost-effective strategy for managing MS in pregnant women compared to discontinuation of treatment at conception or after the first trimester of pregnancy. This approach not only offers significant health benefits to women by reducing relapse rates and slowing disability progression but is also proves economically attractive from the perspective of the UK healthcare system. These findings should inform clinical guidelines and support healthcare providers and women with MS that are planning their family in making evidence-based decisions to improve the management of MS during pregnancy. Furthermore, additional research is essential to refine treatment guidelines and optimise health outcomes for both maternal and foetal wellbeing in the management of MS in pregnancy.

## Supplementary Information

Below is the link to the electronic supplementary material.

Supplementary file1 (DOCX 26 KB)

## Data Availability

Data sharing not applicable to this article as no datasets were generated or analysed during the current study.
